# Modulation of Immune Responses to Influenza A Virus Vaccines by Natural Killer T Cells

**DOI:** 10.3389/fimmu.2020.02172

**Published:** 2020-10-20

**Authors:** John P. Driver, Darling Melany de Carvalho Madrid, Weihong Gu, Bianca L. Artiaga, Jürgen A. Richt

**Affiliations:** ^1^Department of Animal Sciences, University of Florida, Gainesville, FL, United States; ^2^Diagnostic Medicine/Pathobiology, College of Veterinary Medicine, Kansas State University, Manhattan, KS, United States

**Keywords:** natural killer T (NKT) cells, influenza A virus, vaccines, immune modulation, adjuvant

## Abstract

Influenza A viruses (IAVs) circulate widely among different mammalian and avian hosts and sometimes give rise to zoonotic infections. Vaccination is a mainstay of IAV prevention and control. However, the efficacy of IAV vaccines is often suboptimal because of insufficient cross-protection among different IAV genotypes and subtypes as well as the inability to keep up with the rapid molecular evolution of IAV strains. Much attention is focused on improving IAV vaccine efficiency using adjuvants, which are substances that can modulate and enhance immune responses to co-administered antigens. The current review is focused on a non-traditional approach of adjuvanting IAV vaccines by therapeutically targeting the immunomodulatory functions of a rare population of innate-like T lymphocytes called invariant natural killer T (iNKT) cells. These cells bridge the innate and adaptive immune systems and are capable of stimulating a wide array of immune cells that enhance vaccine-mediated immune responses. Here we discuss the factors that influence the adjuvant effects of iNKT cells for influenza vaccines as well as the obstacles that must be overcome before this novel adjuvant approach can be considered for human or veterinary use.

## Introduction

Influenza A viruses (IAVs) are a genetically diverse group of segmented RNA viruses capable of infecting birds and mammals, including swine, bats, and humans (1–3). IAV infections result in a highly contagious acute respiratory disease that is capable of causing substantial morbidity but usually low mortality (4, 5). IAVs are a significant burden to human and animal health and have the potential to occasionally cause pandemics. Vaccination is a cornerstone of IAV mitigation. However, the high genetic/antigenic diversity of IAVs, which is a result of (i) the rapid IAV mutation rates (“genetic drift”) and (ii) the reassortment ability between genetically different IAV strains (“genetic shift”), inherently limits vaccine effectiveness (6). The genetic evolution of IAVs by genetic drift and shift associated with the heterogeneous and complex immune response to influenza vaccines requires annual updates of human IAV vaccines and often results in vaccine failure (2, 7). For example, the 2018–2019 influenza vaccines for humans were reported to have an estimated vaccine effectiveness of 29% (8), based on the relative difference in influenza risk between vaccinated and unvaccinated participants (9). This low effectiveness was due to the circulation of an influenza H3N2 virus which was antigenically drifted from the H3N2 virus isolate included in the vaccine (8). The use of adjuvants that increase the scope, scale, and quality of innate and adaptive immune responses can improve the effectiveness of IAV vaccines significantly (10). Most adjuvants fall into two categories. The first is delivery systems that enhance antigen release, stability, and uptake (11). The second uses immune stimulatory molecules, such as Toll-like receptor ligands, that induce the release of cytokines and chemokines from innate immune cells, which drives antigen-presenting cell (APC) maturation and licensing and, therefore, improves immunogenicity of the respective antigens (12).

Although conventional adjuvants can stimulate cellular and humoral immune responses and reduce the antigen dose required in the vaccine, they seldom improve long-term immunity and cross-reactivity against heterologous (i.e., belong to the same subtype but are genetically different IAV isolates) and heterosubtypic (i.e., belong to different IAV subtypes) IAV strains, which is greatly needed (13). This has led researchers to explore using non-traditional adjuvants to improve vaccine efficacy, including the powerful immunoregulatory effects of innate T lymphocyte populations, such as γδT cells, and CD1- and MR-1-resticted T cells. Unlike the major histocompatibility complex (MHC)-restricted T cells, these cells possess a restricted repertoire of T cell receptors (TCR), perform rapid effector responses, and recognize a limited selection of non-peptide molecules, including small metabolites and lipids (14). Currently, the innate T cell population with the most potential to enhance vaccines is natural killer T (NKT) cells, which recognize lipid and glycolipid ligands presented by the MHC class I-like molecule CD1d (14). These cells have phenotypic characteristics of both T cells and NK cells and express a semi-invariant TCR (14). Upon activation, NKT cells rapidly release large quantities of multiple cytokines and chemokines capable of boosting adaptive immune responses. Importantly, a subset of NKT cells known as type I or invariant NKT (iNKT) cells that express an invariant αβ TCR, can be globally and specifically activated using derivatives of the prototypic antigen known as (2S,3S,4R)-1-*O*-(α-D-galactopyranosyl)-N-hexacosanoyl-2-amino-1,3,4-octadecanetriol, also called α-galactosylceramide (α-GalCer), which was first isolated from a marine sponge (*Agelas mauritianus*) (15, 16). Activation with α-GalCer induces iNKT cells to generate a potent immune response to a wide range of co-delivered antigens.

Since the discovery of α-GalCer, numerous studies have explored how iNKT cell responses can adjuvant vaccines against different infectious diseases [reviewed in (17–20)]. However, most of these studies have used the mouse model of IAV infection, largely because it is well-established and easy to work with. These studies have almost invariably reported that iNKT cells are capable of substantially enhancing the quality and the scale of IAV vaccine responses (21–33). In this review, we summarize the current knowledge about therapeutically harnessing iNKT cell activities to improve IAV vaccines in mice and other animal models. We also address important factors that influence the adjuvant effects of therapeutically activated iNKT cells, which must be considered to safely and effectively exploit the adjuvant potential of iNKT cells for human or livestock vaccines.

## NKT Cell Characteristics

Although the name “natural killer T cell” first appeared in the literature in 1995 (34), these cells were first described in 1987 as a subset of T cells with moderate levels of αβ TCRs and NK1.1, a marker characteristic of natural killer cells (35–38). Over the subsequent decade, it was established that NKT cells express a highly restricted TCR repertoire (39), produce developmentally regulated Th1 and Th2 cytokines (40), bind CD1d as their antigen presenting molecule (41), and recognize glycolipid/lipid ligands (15, 42). It was further discovered that NKT cells can be divided into two functionally distinct classes: type I and type II NKT cells (43). Type I NKT or iNKT cells express a highly restricted TCR repertoire which recognizes CD1d-bound α-GalCer (44, 45). Type II NKT cells, also known as diverse NKT cells, express a less restricted TCR repertoire and recognize different glycolipids than iNKT cells, such as sulfatides (46). This review will discuss iNKT cells as it is this subset which can be therapeutically targeted using glycolipid antigens.

iNKT cells recognize both endogenous and exogenous ligands (42). Their recognition of endogenous ligands enables iNKT cells to interact with inflamed or injured tissues which overexpress lipid molecules (47). Most exogenous iNKT cell antigens are glycolipid and phospholipid components of bacterial cell walls, such as mycobacterial phosphatidylinositolmannosides and monoglycosylceramides from gram-negative bacteria (42, 48). iNKT cells also respond to antigens from protozoan parasites, including phospholipids from *Leishmania* and glycophosphatidylinositol from *Plasmodium* and *Trypanosoma* (49, 50). iNKT cells in most species react to α-GalCer and its synthetic analog KRN7000 (51–53). These molecules have been widely used to study iNKT cell function since they strongly activate these cells. α-GalCer stimulated mouse iNKT cells produce a wide variety of cytokines, including IFN-γ, IL-2, IL-3, IL-4, IL-5, IL-9, IL-10, IL-13, IL-17, IL-21, IL-22, and tumor necrosis factor (TNF)-α and -β (54–57). Stimulated mouse iNKT cells also secrete chemokines, including RANTES (regulated on activation, normal T cell expressed and secreted), monocyte chemoattractant protein (MCP)-1, eotaxins, and macrophage inflammatory protein (MIP)-1α and MIP-1β (58–61). Many of these cytokines modulate cellular and humoral immune responses against foreign antigens, which is why α-GalCer activated iNKT cells can enhance the scale and the scope of vaccine responses against a wide variety of pathogens.

## iNKT CELL-CD1d System in Mammals

The defining feature of iNKT cells is the expression of a TCR with an invariant Vα chain rearrangement and limited Vβ chain usage. Mouse iNKT cells express a single α chain (Vα14-Jα18) that is paired with a limited number of Vβ chains (Vβ2, Vβ7, or Vβ8.2) (39, 62, 63). Rats use a homologous Vα14-Jα18 rearrangement paired with Vβ8.2 chains but have four Vα14 genes with differential tissue expression (64). The human invariant receptor is composed of a Vα24-Jα18 rearrangement paired with Vβ11 (39, 65, 66), while the porcine iNKT TCR is composed of a Vα10-Jα18 chain paired with a Vβ25-chain, both of which are highly homologous to the human Vα24-Jα18 and Vβ11 TCR chains (67). A consequence of the remarkably conserved nature of the TCR-CD1d system is that CD1d tetramers often cross-react among different animal species. For instance, human CD1d tetramers cross-react with mouse iNKT cells and *vice versa* (45), and both mouse and human CD1d tetramers cross-react with pig iNKT cells (68). Interestingly, rat iNKT cells are only partially identified by mouse CD1d tetramers and require the use of rat CD1d molecules in glycolipid-loaded tetramers (69). Overall, the CD1d-mediated recognition of α-GalCer by iNKT cells is highly conserved through mammalian evolution (70). This has the advantage that many aspects of glycolipid therapy research in preclinical mouse models can be directly translated to target animal species, including humans.

Not all mammals harbor CD1d genes in their genomes, and some that do, do not express functional transcripts and/or CD1d proteins that are capable of interacting with iNKT cells. Humans (71), primates (72, 73), mice (15), rats (64), cotton rats (74), pigs (75, 76), and dogs (77) have been reported to possess functional iNKT cell-CD1d systems and iNKT cells that react to α-GalCer. Ruminants were thought to harbor two copies of *CD1d* that are pseudogenes (*CD1d1* and *CD1d2*) due to a mutated start codon and a first intron that cannot be translated into functional proteins (78, 79). However, it was later discovered that the bovine *CD1d1* gene has an alternative start codon that produces CD1d proteins capable of being expressed on the cell surface (80). Interestingly, the antigen binding site in bovine CD1d1 is smaller than in human and mouse CD1d proteins, which prohibits α-GalCer from binding. Instead, bovine CD1d1 appears to present glycolipids with shorter alkyl chains than α-GalCer (80, 81). The sequences of the equine iNKT invariant α-chain TCR and CD1d have conserved residues that align with their human and mouse counterparts. Nevertheless, equine iNKT cells have yet to be isolated and horses do not respond to synthetic glycolipids that activate iNKT cells in other species (82).

## Mechanisms of iNKT Cell Activation

iNKT cells can be directly activated by TCR signaling after engaging CD1d-bound glycolipid antigens, or indirectly via cytokines from pathogen recognition receptor-stimulated APCs. Indirect activation sometimes involves weak TCR signals from low-affinity microbial or self-lipid antigens but can also occur in the absence of TCR stimulation (83–88). Directly activated mouse iNKT cells secrete a mixture of Th1 and Th2 cytokines, which differs from iNKT cells indirectly activated through pro-inflammatory cytokines that mainly produce Th1-type cytokines (89, 90). The variety and the quantity of cytokines produced by directly activated iNKT cells depend on the strength of the interactions between the iNKT TCR and the lipid-CD1d complex (43, 83, 91, 92). However, additional factors, including different iNKT cell subsets, the half-life of TCR-ligand binding, ligand density, and the uptake and presentation of iNKT cell-activating glycolipids by APCs, may play a role in determining their cytokine bias (93–97). α-GalCer strongly activates iNKT cells, which induces the rapid upregulation of T cell activation markers and the secretion of several cytokines, especially IFN-γ, IL-2, and IL-4 within hours after stimulation. α-GalCer activated iNKT cells also proliferate and may expand up to 10-fold in some organs by 4 days post-treatment, after which they contract to baseline levels (98, 99). Unlike conventional T cells, iNKT cells do not possess memory functions. In fact, secondary α-GalCer administration actually results in a significantly weaker iNKT cell response compared to primary stimulation with this antigen, characterized by reduced proliferation and cytokine secretion (100, 101). This hyporesponsive state lasts for at least 1 month after initial activation (101–103). The same hyporesponsive state has been reported in mice challenged with bacterial pathogens (104–106), toxins (107), and TLR agonists (104). The response of iNKT cells to α-GalCer have been studied most extensively in mice. However, *in vivo* studies in humans, chimpanzees, macaques, swine, and cotton rats have found that α-GalCer can stimulate iNKT cell activities in a wide variety of mammals.

iNKT cells indirectly activated by APCs participate in immune responses against numerous microorganisms that lack cognate lipid antigens. Various pathogen-associated molecular patterns (PAMPs) and some danger-associated molecular patterns (DAMPs) have been shown to induce iNKT cell-activating cytokines (108–112), such as type I interferons, IL-12, IL-18, and IL-33 (88, 90, 113–115). Interleukin-12 signaling appears to be particularly important as iNKT cells express high baseline levels of the IL-12 receptor (116). Many bacterial and viral infections stimulate sufficient IL-12 to activate iNKT cells with low-affinity endogenous ligands or without TCR signaling (88, 89, 113, 117, 118). Indirect iNKT cell activation may also be induced by IFN-α and IFN-β from TLR-7- and TLR-9-activated APCs (113) or by a combination of IL-12 and IL-18, which are produced by TLR-4- and TLR-9-stimulated APCs (88, 90, 114). iNKT cells can also be activated *via* their NK receptors that provide both activation and regulation signals in response to stress-induced ligands (119–121). Indirectly activated iNKT cells develop distinct effector functions compared to directly activated iNKT cells. One important difference is that a large fraction of cytokine-activated iNKT cells acquire the ability to express perforin that may allow them to carry out cytolytic functions *in vivo* (54). Furthermore, indirectly activated iNKT cells secrete large quantities of IFN-γ without IL-4, whereas directly activated iNKT cells often produce both cytokines simultaneously (122). The weak TCR signaling that occurs during indirect iNKT cell activation promotes IFN-γ production by inducing histone H4 acetylation near the IFN-γ locus. This enables iNKT cells to produce IFN-γ upon subsequent exposure to IL-12 and IL-18 without concurrent TCR stimulation (114). Unlike during direct activation, iNKT cells remain motile during stimulation with cytokines, which may enable them to disseminate IFN-γ as they migrate, amplifying their impact on immune responses (114). It is likely that IAV vaccines trigger several indirect activation-mediated iNKT cell effector functions and that some of these responses will support (or perhaps counteract) the direct activation effects of co-delivered glycolipid antigens. In this scenario, achieving optimal IAV vaccine immunity will require studies to evaluate combining different dosages of iNKT cell agonists and IAV vaccines.

## Helper Functions of iNKT Cells

**Figure 1:**
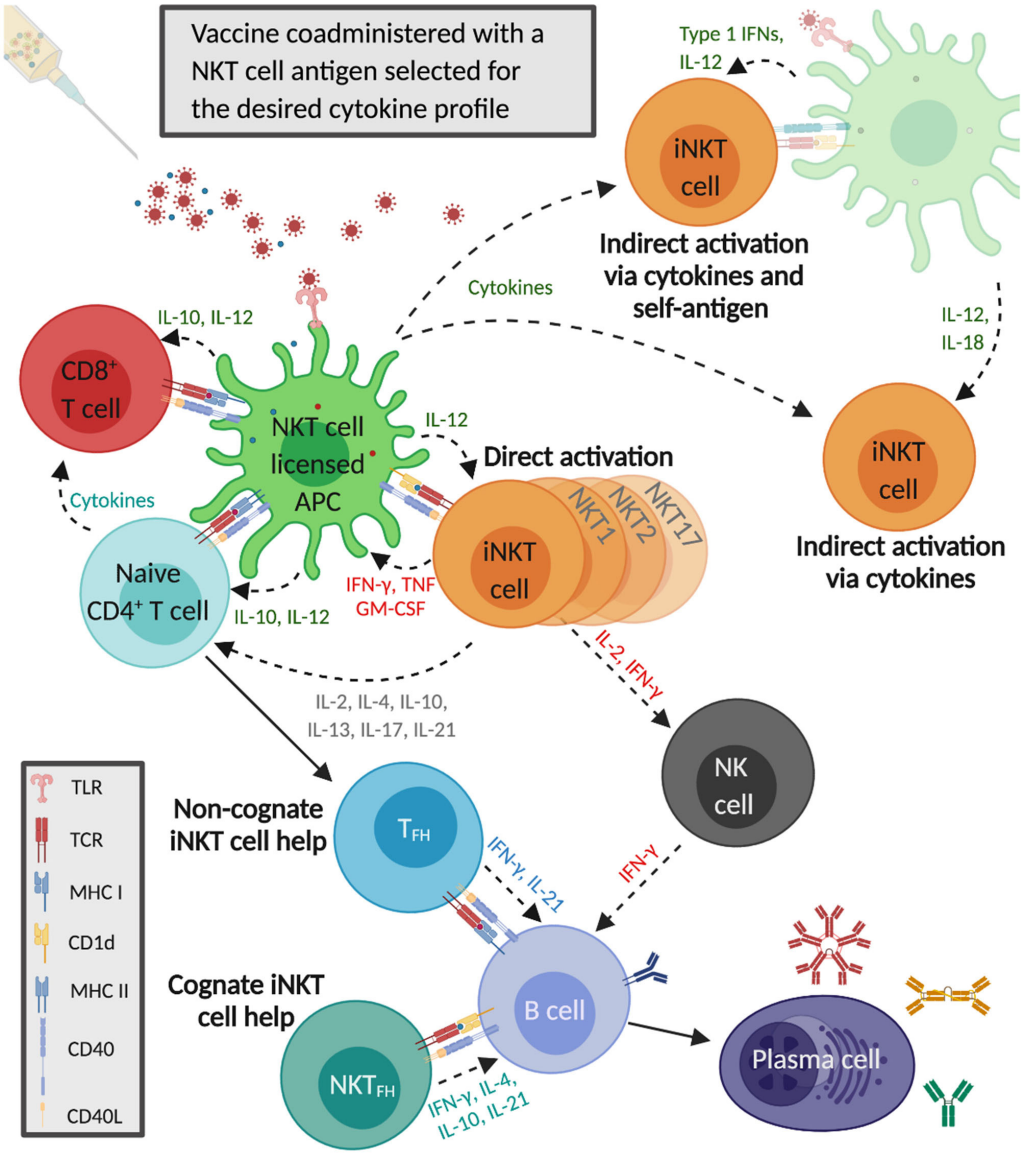
Invariant natural killer T cells activate and regulate innate and adaptive immune responses that enhance influenza A virus (IAV) vaccine immunity. The co-administered IAV vaccine antigens and the selected α-GalCer analog are internalized by dendritic cells that simultaneously present the α-GalCer analog on CD1d to iNKT cells and IAV epitopes on major histocompatibility complex (MHC) II to CD4^+^ T cells. Cytokines secreted by iNKT cells increase MHC class II and CD40 presentation to naïve CD4^+^ T cells, which generates follicular T helper cells (T_FH_) that provide non-cognate help to B cells. iNKT cells also undergo indirect activation in response to proinflammatory cytokines released by IAV-stimulated antigen-presenting cells (APCs), with or without engagement of the iNKT TCR by CD1d-presented self-antigens. iNKT cell-licensed dendritic cells (DCs) generate vaccine-specific CD8^+^ T cells which become further activated by cytokines secreted by α-GalCer and IAV-stimulated APCs as well as iNKT cells and CD4^+^ T cells. The iNKT cells recognizing α-GalCer on DCs differentiate into iNKT follicular helper (iNKT_FH_) cells that provide cognate help to B cells specific for vaccine antigens. iNKT cells also boost humoral immunity by trans-activating natural killer cells that can stimulate B cells to secrete immunoglobulin G.

iNKT cells are capable of generating immune responses that in many ways mirror conventional CD4^+^ T cell help (Figure 1). Activated iNKT cells induce APCs to mature when they engage antigen-bound CD1d on their surface (123). These iNKT cell-conditioned APCs, in turn, produce cytokines and T cell costimulatory molecules that further prime iNKT cells, causing them to upregulate CD40L, and secrete IFN-γ and granulocyte-macrophage colony-stimulating factor. This induces surrounding APCs to mature and activate additional iNKT cells (113, 123, 124). iNKT cell-licensed APCs prime conventional CD4^+^ T cells against co-delivered peptide antigens, which results in enhanced cytotoxic CD8^+^ T cell responses and induces CD4^+^ T cells to become follicular helper T (T_FH_) cells. iNKT cell-conditioned APCs also acquire the ability to cross-present peptide antigens to CD8^+^ T cells (125–127). The mechanisms through which iNKT cells enhance humoral immune responses have been extensively reviewed (128–132). One mode involves iNKT cells directly interacting with B cells presenting glycolipid ligands on CD1d, which is referred to as cognate B cell help. Initially, iNKT cells recognize glycolipid antigens on dendritic cells (DCs) and differentiate into iNKT follicular helper (iNKT_FH_) cells that adopt a phenotype similar to T_FH_ cells (133, 134). These iNKT_FH_ cells then activate B cells specific for protein antigens through a process that requires CD1d expression by B cells, B7-1/B7-2, and CD40 ligation by the iNKT cells and the secretion of IFN-γ and IL-21. Cognate B cell help stimulates plasmablast expansion, germinal center formation, antibody class switching, and moderate affinity maturation (135, 136). iNKT cells can also provide non-cognate B cell help which occurs when iNKT cells indirectly activate B cells by inducing T_FH_ cells specific for protein antigens displayed by B cells (137). This mode of activation is thought to drive enhanced antibody production when α-GalCer is co-administered with immunizing antigens; protein and α-GalCer are internalized by DCs that simultaneously present peptide fragments of the protein antigen on MHC class II to naïve CD4+ T cells and α-GalCer on CD1d to iNKT cells. The T_FH_ cells that result provide CD1d-independent antigen-specific help for the proliferation of B cells in germinal centers, antibody class switching, affinity maturation, and the generation of plasma cells and memory B cells. In addition, activated iNKT cells trans-activate NK cells to produce large quantities of IFN-γ that stimulate B cells to secrete IgG (138). Non-cognate B cell help is probably important after virus exposure when iNKT cells may shape B cell responses by producing an early wave of IL-4 that seeds germinal centers and activates antigen-experienced B cells (139).

The benefit of eliciting T helper responses via iNKT cells compared to CD4^+^ T cells is that iNKT cells constitute a much greater fraction of total T cells than any antigen-specific CD4^+^ T cell clone (140, 141). Furthermore, iNKT cells can be globally and specifically activated using α-GalCer analogs due to the highly non-polymorphic CD1d molecule (67, 78, 142). Conversely, conventional CD4^+^ T cells are restricted by the high level of inter-individual MHC class II polymorphism, which limits the efficacy of peptide-based vaccines in outbred populations. Co-administering iNKT cell ligands with vaccines that induce a wide array of conventional T helper cell responses has the potential to induce wide-ranging cellular and humoral immune responses capable of greatly improving the durability and the cross-protection of vaccines, including against IAVs.

## Role of iNKT Cells in Immunity to IAV Infections

Mice lacking iNKT cells are more susceptible to IAV infections than iNKT cell-intact mice (143–146), indicating that these cells contribute to IAV immunity. iNKT cell activation is likely through the indirect pathway as IAVs contain no known iNKT cell ligands. Nevertheless, stimulation may be enhanced by interactions with CD1d-bound endogenous glycolipids which often increase after viral infection (147). iNKT cells migrate to the lungs during the early stages of IAV infections, alongside the recruitment of neutrophils and the rapid induction of pro- and anti-inflammatory cytokines that prime immune cells, including iNKT cells (146, 148). Mouse studies have reported that airway-resident iNKT cells prevent virus replication and limit lung damage through a combination of (i) reducing the suppressive capacity of myeloid-derived suppressor cells that inhibit influenza-specific immune responses (143), (ii) activating lung-resident NK cells (145), and (iii) directly lysing IAV-infected monocytes (144). In addition, iNKT cells stimulated by IL-1β and IL-23 produce large amounts of IL-22 that protects the lung epithelium from influenza-mediated damage (149). iNKT cells have also been investigated for their role in shaping pre-existing immunity against re-infections with the same or heterologous influenza viruses. Benton et al. showed that CD1d knockout mice previously infected with A/Puerto Rico/8/34 (H1N1) or A/Philippines/2/82/X-79 (H3N2) and re-infected after 4 weeks with the correspondent homologous or heterologous H1N1 or H3N2 viruses were just as susceptible to re-infection as wildtype mice (150). These findings suggest that iNKT cell responses might be superfluous for generating immune memory or cross-protection after a natural IAV infection. This contrasts with other studies showing that therapeutically activated iNKT cells improve pre-existing immunity from IAV vaccination and suggests that immunity generated by iNKT cells might differ depending on whether they are activated indirectly during an IAV infection or *via* glycolipid antigens.

## Studies Using iNKT Cell Agonists With IAV Vaccines

At least 17 publications have reported the effects of iNKT cell agonists for adjuvanting IAV vaccines (Table 1). Most of these studies have used mice because they are relatively inexpensive and compatible with a wide variety of vaccine formats. However, swine and non-human primates [pigtail macaques (*Macaca nemestrina*)] have also been tested. To our knowledge, Ko et al. were the first group to explore adjuvanting IAV vaccines with iNKT cell agonists (26). They reported that BALB/c mice intranasally co-administered with three doses (1 week apart) of α-GalCer and HA antigen derived from the mouse-adapted A/PR/8/34 (PR8, H1N1) IAV were much better protected from a lethal dose of the homologous virus than mice immunized with vaccine alone. α-GalCer induced greater levels of mucosal and systemic IgA and IgG antibodies. The same group later demonstrated that a single intranasal vaccination with inactivated PR8 and α-GalCer was sufficient to induce long-lasting PR8-specific IgG and IgA that protected mice from PR8 infection 3 months after the vaccination (22).

**Table 1:** Summary of studies on modulating immune responses to influenza A virus (IAV) using invariant natural killer T (iNKT) cell agonists.

**Animal model**	**Vaccination**		**Vaccine format**	**iNKT cell agonist (dose per animal)**	**Mode of action**	**References**
	**Route**	**Strain/subunit**				
Mouse (BALB/c)	i.n.	H1N1 PR8	Immunization with PR8 HA antigen with α-GalCer three times at 1-week intervals. Infection with 20 LD_50_ PR8 2 weeks after final immunization.	α-GalCer (0.125, 0.5, 2 μg)	α-GalCer induced mucosal secretory IgA as well as systemic IgG antibody responses against virus-derived antigen and reduced clinical signs.	(26)
Mouse (BALB/c)	i.n.	H1N1 PR8	Immunization with inactivated PR8 with α-GalCer. Infection with 20 LD_50_ PR8 2 weeks and 3 months after immunization.	α-GalCer (0.5 μg)	α-GalCer induced both mucosal and systemic antibody responses, provided protective immunity against challenge with live PR8 and induced cytotoxic CD8^+^ T cells.	(22)
Mouse (BALB/c)	i.n./i.m.	H1N1 PR8 H1N1A/Yamagata H3N2A/Guizhou B/Ibaraki	Immunization with PR8, A/Yamagata, A/Guizhou, or B/Ibaraki HA vaccine with α-GalCer twice at 4 weeks apart. Infection with 40 LD_50_ PR8 2 weeks after the second immunization.	α-GalCer (2 μg)	i.n., not i.m., vaccination (PR8 and A/Yamagata) with α-GalCer boosted IgA and IgG and cross-protection against heterosubtypic virus infection.	(23)
Mouse (BALB/c)	i.n.	H1N1 PR8	Immunization with PR8 with α-GalCer twice at 4 weeks apart. Infection with 40 LD_50_ A/Yamagata, A/Guizhou, or B/Ibaraki 2 weeks after the second immunization.	α-GalCer (2 μg)	i.n. vaccination with α-GalCer protected against challenge with homologous (A/PR8) and heterologous (A/Yamagata) viruses.	(23)
Mouse (BALB/c)	i.n.	H5N1 NIBRG14	Immunization with NIBRG14 (H5N1) inactivated vaccine with α-GalCer twice, 4 weeks apart. Infection with 10^3^ PFU of A/Vietnam (H5N1) or A/HK483 (H5N1) influenza virus 2 weeks after the second immunization.	α-GalCer (2 μg)	i.n. vaccination with α-GalCer increased nasal IgA and serum IgG and induced cross-protection against H5N1 influenza infection.	(23)
Mouse (BALB/c)	i.n.	H1N1 rNS1 1-73	Immunization with live attenuated rNS1 1-73 with different amounts of α-C-GalCer. Infection with 100 LD_50_ PR8 3 weeks after the immunization.	α-C-GalCer (0.11, 0.33, 1, 2, 3, 4 μg)	α-C-GalCer used between 0.1 and 1 μg per mouse reduced mortality and morbidity. The adjuvant also increased the amount of influenza virus-specific total IgG, IgG1, and IgG2a antibodies as well as IFN-γ secreting CD8^+^ T cells.	(27)
Mouse (BALB/c)	i.n.	H1N1 PR8	Immunization with inactivated PR8 with α-GalCer or α-GalCer analogs. Infection with 5 LD_50_ PR8 4 weeks after the immunization or 100 LD_50_ PR8 5 weeks after immunization.	α-GalCer (0.5 μg); KBC-007 (0.5 μg); KBC-009 (0.5 μg)	Co-immunization with α-GalCer, KBC-007 and KBC-009 increased PR8-specific systemic IgG and mucosal IgA. α-GalCer and KBC-009 (but not KBC-007) increased antigen-specific lymphocyte proliferation, cytokine production, and cytotoxic CD8^+^ T cell activity and induced complete protection from live virus infection.	(32)
Mouse (C57BL/6)	i.v.	H1N1 PR8-OVA257	Immunization with SLP-conjugated vaccine PR8-OVA257 with α-GalCer. Infection with 10^4^ PFU HKx31-OVA257 6–8 weeks after the immunization.	α-GalCer (76 ng)	α-GalCer-peptide conjugates induced OVA-specific T cell responses and protected against IAV infection.	(28)
Mice (C57BL/6)	i.m.	HA/NA from H3N2 PNM07	Immunization with PNM07 protein and α-GalCer twice at 2 weeks apart.	α-GalCer (0.1 μg)	Immunization with H3N2 PNM07 plus α-GalCer increased titers of H3N2-specific antibodies.	(30)
Mouse (C57BL/6)	i.m.	HA/NA from H1N1 NC20	Immunization with NC20 protein with α-GalCer twice at 2 weeks apart. Infection with 100 LD_50_ H1N1 A/WS/33 2 weeks after the second immunization.	α-GalCer (0.1 μg)	α-GalCer increased the survival rate after challenge.	(30)
Mouse (C57BL/6)	i.m.	H3N2 PNM07	Immunization with H3N2 PNM07 protein with α-GalCer twice at 0 and 2 weeks, boosted with PNM07 at 30 weeks.	α-GalCer (0.1 μg)	Immunization with H3N2 PNM07 plus α-GalCer resulted in a higher antibody response and increased expansion of the antigen-specific memory B cells.	(30)
Mouse (C57BL/6)	s.c.	H1N1 PR8	Immunization with inactivated PR8 with α-GalCer. Infection with 10^4^ PFU of live H3N2 HKx31 6 weeks after immunization.	α-GalCer (1 μg)	Vaccination with α-GalCer increased the survival of long-lived memory cytotoxic CD8^+^ T cell populations capable of boosting protection against heterologous IAV challenge.	(24)
Mouse (BALB/c)	i.m.	pCHA5 for H5N1	Immunization with pCHA5 with C34 twice at 3 weeks apart. Infection with 200 LD_50_ NIBRG14 (a reassortant H5N1 virus) 2 weeks after the immunization.	C34 (2 μg)	C34 increased titers of HA-specific antibodies and T cells and improved survival after challenge.	(31)
Mouse (C57BL/6)	i.v.	H1N1 PR8	Immunization with PR8 HA mRNA-transfected CD1d-allogeneic cells loaded with α-GalCer (aAVC-HA). Infection with 10^3^ PFU PR8 2 weeks after the immunization.	5 × 10^5^ aAVC-HA precultured with α-GalCer (500 ng/mL)	Vaccination with aAVC-HA preserved body weight, increased survival after infection, and increased titers of HA-specific IgG.	(29)
Mouse (BALB/c)	i.p.	M2e peptide	Immunization with M2e peptide with α-GalCer twice at 3 weeks apart. Infection with 10^3^ PFU H5N1 3 weeks after the final immunization.	α-GalCer (1 μg)	α-GalCer co-administered with M2e peptide reduced morbidity, mortality and up-regulated IFN-γ, IL-4 after challenge.	(25)
Mouse (BALB/c)	i.m.	DNA vaccine encoding M2	Immunization with DNA vaccine encoding M2 with α-GalCer three times at 2-week intervals. Infection with 1 LD_90_ PR8 2 weeks after the final immunization.	α-GalCer (1 μg)	α-GalCer increased M2-specific IgG; lymphocyte proliferation; IFN-γ and IL-12 and IL-4 production; and survival rate after virus challenge.	(21)
Mouse (BALB/c)	i.m.	H1N1 CA07, A/Hong Kong (H3N2), B/Phuket, and B/Texas	Immunization with split influenza HA vaccine with 7DW8-5 twice at 2-week intervals. Infection with 10 MLD_50_ H1N1 CA04 3 weeks after the final immunization.	7DW8-5 (1 μg or 10 μg)	7DW8-5 was sufficient to protect the mice from lethal infection but did not completely prevent virus replication.	(33)
Pig	i.n.	H1N1 OH07	Immunization with inactivated SwIV OH07 with α-GalCer once. Infection with homologous SwIV OH07 (10^6^ TCID_50_ per pig) 3 weeks after the immunization.	α-GalCer (50 or 250 μg)	α-GalCer increased IAV-specific mucosal IgA and upregulated the expression of BAFF.	(151)
Pig	i.n.	H1N1 OH07	Immunization with inactivated SwIV OH07 with α-GalCer once. Infection with homologous SwIV OH07 (10^6^ TCID_50_ per pig) 3 weeks after the immunization.	α-GalCer (50 or 250 μg)	α-GalCer (250 μg) administration reduced pulmonary viral load and increased SwIV-specific IgA secretion both in the lungs and the airways.	(152)
Pig	i.m.	H1N1 CA04	Immunization with inactivated H1N1 CA04 with α-GalCer twice at 16-day intervals. Infection with 10^6^ TCID_50_ CA04 16 days after the immunization.	α-GalCer (100 μg/kg)	Vaccination with α-GalCer enhanced both systemic and mucosal influenza-specific antibodies and inhibited viral replication in the upper and the lower respiratory tracts.	(153)
Pigtail macaques	i.v.	Live-attenuated IAV encoding three distinct SIV epitopes (flu-SIV)	A single dose of α-GalCer pulsed onto whole blood for 2 h and re-infused with flu-SIV; additional vaccinations without α-GalCer on days 28, 56, and 119.	α-GalCer (5 μg)	α-GalCer reduced vaccine-specific CD8^+^T cells and had no effect on the frequency of iNKT cells or IAV-specific antibodies; reduced influenza-specific CD8^+^ T cells.	(72)

*PR8, H1N1 strain A/Puerto Rico/8/34; NC20, H1N1 strain A/New Caledonia/20/99; A/WS/33, H1N1 strain A/Wilson-Smith/1933; PNM07, H3N2 strain A/Panama/2007/99; A/Yamagata, H1N1 strain A/Yamagata/120/86; A/Guizhou, H3N2 strain A/Guizhou/54/89; B/Ibaraki, B/Ibaraki/2/85; rNS1 1-73, a PR8 mutant virus expressing only the first 73 amino acids in the NS1 gene; NIBRG-14, reassortant virus derived from PR8 and A/Vietnam/1194/2004 (H5N1) virus (in which the polybasic HA cleavage site has been excised); M2e, ectodomain of M2 protein; CA07, A/California/07/2009 (H1N1) virus; A/Hong Kong, A/Hong Kong/4801/2014 (H3N2) virus; B/ Phuket, B/Phuket/3073/2013 (Yamagata lineage) virus; B/Texas, B/Texas/2/2013 (Victoria lineage) virus; OH07, a zoonotic SwIV H1N1 (Sw/OH/24366/07); CA04, H1N1pdm09 strain A/California/04/2009; α-C-GalCer, alpha-C galactosylceramide; α-GalCer, alpha-galactosylceramide; 7DW8-5, 4-fluorophenylundecanoyl-alpha-galactosylceramide; aAVC-HA, HA-expressing artificial adjuvant vector cells; i.m., intramuscular; i.n., intranasal; i.p., intraperitoneal; s.c., subcutaneous; i.v., intravenous; WB, whole blood*.

iNKT cell agonists have also been assessed for their ability to improve vaccine-mediated cross-protection against heterologous and heterosubtypic virus infections. Kamijuku et al. administered α-GalCer to BALB/c mice in combination with HA-based vaccines derived from a variety of IAV strains. Strong protection was induced against heterologous IAV strains within the same HA vaccine subtype and partial protection was generated against the heterosubtypic strains (23). A follow-up study demonstrated that an inactivated whole-virion vaccine of H5N1 IAV generated robust cross-protection against a heterologous H5N1 IAV strain when administered intranasally with, but not without, α-GalCer. The cross-protective effects of iNKT cell activation were found to be mediated by mucosal IgA production and effector responses that require IL-4, but not IFN-γ (23).

Other strategies that have been tested include a study where the α-GalCer derivative α-C-galactosylceramide (α-C-GalCer) enhanced the immune response elicited by a live attenuated A/PR/8/34 virus expressing only the first 73 amino acids in the NS1 gene; NS1 is needed to inhibit critical innate host immune factors (27). BALB/c mice were co-administered the live attenuated virus (LAV) vaccine with different doses of α-C-GalCer ranging from 0 to 4 μg/mouse. Interestingly, only mice that received low doses of α-C-GalCer survived, while mice treated with the 4 μg dose or the LAV vaccine alone were not protected. This finding suggests that activating iNKT cells too strongly may be detrimental for LAV vaccine applications since their limited replication capacity might be abolished by iNKT cell-mediated innate immune responses before they have had an opportunity to induce vaccine-specific immunity. α-GalCer has also been used to adjuvant the normally poorly immunogenic IAV M2 ectodomain (M2e); BALB/c mice co-immunized with M2e and α-GalCer were fully protected against a highly pathogenic H5N1 avian IAV infection and exhibited significantly reduced morbidity and lung viral titers compared to mice that were immunized without α-GalCer (25). The enhanced protection was associated with augmented IgG1 and IgG2 antibody levels and greater IFN-γ and IL-4 upregulation after infection. Another study tested the efficacy of α-GalCer-peptide conjugated vaccines composed of synthetic long peptides (SLP) containing an immunogenic peptide covalently attached to α-GalCer by a cleavable linker (28). This ensures that the vaccine peptide and α-GalCer are delivered to the same APC and enables iNKT cells to license the same APCs involved in stimulating conventional CD4^+^ and CD8^+^ T cell responses against the vaccine antigen. C57BL/6 mice were vaccinated with α-GalCer conjugated SLPs composed of an immunogenic peptide of chicken ovalbumin and challenged 6 weeks later with a recombinant ovalbumin expressing IAV. The SLP vaccine provided much greater protection than previous infection with the backbone virus. However, it remains to be determined whether this approach provides protection against non-OVA-expressing IAVs. Another strategy used an “adjuvant vector cell” (aAVC) system comprised of CD1d^+^ HA mRNA-transfected cell lines (NIH3T3 for mice and HEK293 cells for humans) loaded with α-GalCer. C57BL6/J mice immunized with aAVC-HA were protected from a lethal dose of PR8 2 weeks later. The efficacy of this approach seemed to depend on the formation of germinal centers and T_FH_ cells and was more effective than a co-administration of free antigen and α-GalCer (29).

Many articles reporting the adjuvant activities of iNKT cells for IAV vaccines have used the intranasal delivery route of administering α-GalCer because of the importance of this site for pulmonary immunity. α-GalCer administered by this route remains localized to the nasal-associated lymphoid tissues and cervical lymph nodes where it becomes concentrated in the intracellular vesicles of DCs. These DCs co-localize with iNKT cells that accumulate in these tissues through a process that requires the chemokine receptor CXCR6 and its ligand CXCL16 (23). α-GalCer can also induce effective immunity when delivered to tissues beyond the site of infection. For instance, Galli et al. showed that mice immunized *via* the intramuscular route with α-GalCer admixed with HA/NA subunits from human influenza viruses generated antibody titers that were 1–2 logs higher than mice immunized with protein alone and greatly enhanced survival after a lethal IAV infection (30).

iNKT cell responses have also shown promise for generating cross-reactive CD8^+^ T cells against serologically distinct IAV subtypes, which is a major shortcoming of current IAV vaccines. Guillonneau et al. examined cytotoxic CD8^+^ T cell responses in C57BL/6 mice subcutaneously immunized with an inactivated PR8 vaccine, with and without α-GalCer, that were infected with a heterosubtypic H3N2 IAV 6 weeks later (24). iNKT cell activation enhanced the survival of long-lived memory cytotoxic CD8^+^ T cells capable of clearing virus from the lungs while paradoxically diminishing acute phase cytotoxic T cell responses through iNKT cell-dependent production of indoleamine 2,3-dioxygenase, an immune suppressive enzyme (24). The induction of long-lasting CD8^+^ T cells was associated with the upregulation of *Bcl-2*, which is a pro-survival gene. The adjuvant effects of iNKT cells have also shown potential to improve DNA-based vaccines, which stimulate only modest immunity in humans. Two studies have demonstrated that vaccinating mice with α-GalCer derivatives and DNA vaccines encoding an HA consensus sequence of an H5N1 IAV or the IAV M2 protein induces M2-specific cellular and humoral immune responses and protection from virus challenge (21, 31).

Although mouse models have demonstrated that iNKT cell activities can greatly enhance IAV vaccines, it remains unclear whether the same approach would be successful in humans as mice are not natural IAV hosts and mouse and human iNKT cells differ considerably in frequency, subsets, and tissue distribution. Therapeutically activating iNKT cells failed to improve anti-IAV cellular and humoral immune responses when attempted in pigtail macaques, which are considered a good translational model for human IAV infections (72). Nevertheless, we previously reported that α-GalCer substantially increased the efficacy of a killed pandemic 2009 H1N1 IAV vaccine in pigs that were challenged with the homologous virus. Protection was associated with higher levels of vaccine-specific antibodies and T cells and reduced viral replication in the upper and lower respiratory tract compared to pigs that received the vaccine alone (153). Similar results were obtained in studies by Dwivedi et al. and Renu et al., who showed that intranasal co-administration of α-GalCer with UV-inactivated H1N1 vaccines increased mucosal IgA levels, upregulated lung expression of the B cell activation factor BAFF, and substantially reduced virus loads in pigs (151, 152). Collectively, these results are encouraging as swine and human iNKT cells share several key characteristics, and like humans, pigs are natural hosts of IAVs. Furthermore, there may also be potential to use iNKT cell antigens for vaccines against swine influenza and other pig pathogens.

## Factors That Influence the Adjuvant Potential of iNKT Cells

Studies in mice have established that the effects of iNKT cell activation are influenced by a variety of interacting parameters, which should be considered when attempting to use these agents to adjuvant vaccines. Some of the main factors are discussed below.

### iNKT Cell Subsets

The iNKT cell compartment consists of multiple subsets that play distinct roles during pathogen-host interactions and which produce different effector functions after glycolipid stimulation. Subset development occurs through a linear differentiation process characterized by sequential changes in surface markers and transcription factors. Human iNKT cells develop into CD4^−^ or CD4^+^ subsets that secrete Th1 or a mixture of Th1 and Th2 cytokines upon activation, respectively (54, 154). However, these differences in cytokine production are less apparent for mouse iNKT cells (85). Another difference is that the CD4^+^ subset of iNKT cells predominates in mice (155), while human iNKT cells are mostly CD4^−^ (54, 156, 157). Like humans, iNKT cells in pigs are mainly CD4^−^ (52, 68, 75, 153), and both humans and pigs contain a subset of CD8^+^ iNKT cells which is absent in mice (54, 68, 75, 156, 157).

Mouse iNKT cell subsets can be classified according to their development lineages that are acquired during thymic selection (Table 2). These are composed of the three major functionally distinct subsets, NKT1, NKT2, and NKT17, which express the master transcription factors T-bet, GATA-3, and RORγt that also, respectively, engender the fate of Th1, Th2, and Th17 T helper cell subsets as well as ILC1, ILC2, and ILC3 innate lymphoid cells (95, 158–161, 163–165). Each iNKT subset expresses different levels of the transcription factor PLZF, which is critical for the development and the innate functions of iNKT cells (168–170). Additional iNKT cell subsets that differentiate extrathymically have been identified, including NKT_FH_ and NKT10 cells. NKT_FH_ cells are characterized by the expression of Bcl-6 and the secretion of IL-21 and are located in the germinal centers of lymphoid organs. NKT_FH_ cells provide help to B cells during the formation of germinal centers and drive the affinity maturation of antibodies toward lipid antigens (134, 135). NKT10 cells are equivalent to type-1 regulatory T cells (T_REGS_) and produce IL-10 and IL-2. This subset induces the differentiation of anti-inflammatory macrophages and provides help to T_REGS_. Instead of expressing PLZF like other NKT cells, NKT10 express E4BP4, a transcription factor associated with IL-10 production (166).

**Table 2:** Key characteristics of the main invariant natural killer T (iNKT) cell subsets.

**iNKT cell subset**	**Transcription factor**	**Signature cytokines**	**Tissue distribution**	**References**
NKT1	T-bet, PLZF^low^	IL-4, IFN-γ	Liver, spleen, lung, small intestine	(56, 95, 158–162)
NKT2	GATA3, PLZF^hi^	IL-4, IL-5, IL-13	Lung, spleen, mesenteric lymph nodes	(56, 159–163)
NKT17	RORγt, PLZF^int^	IL-17, IL-22	Lymph nodes, lung, skin	(56, 159–162, 164, 165)
NKT_FH_	Bcl-6	IL-21	Germinal centers of lymphoid organs	(134, 135)
NKT10	E4BP4, PLZF^−^	IL-10, IL-2	Adipose tissue	(166, 167)

Individual iNKT cell subsets differentially accumulate in lymphoid and non-lymphoid tissues (171). In mice, most liver iNKT cells are NKT1 cells, while NKT2 cells predominate in the lung, spleen, and mesenteric lymph nodes (56, 162, 171). NKT17 cells are most plentiful in the lymph nodes, lung, and skin (56, 162, 171), while NKT10 cells preferentially accumulate in adipose tissue (167, 171) and NKT_FH_ cells localize in germinal centers, especially in the spleen (134, 135). The effector functions of each subset are distinct and range from NKT1 cells that stimulate proinflammatory responses capable of suppressing cancer and infectious agents (83, 172) to tolerogenic NKT2 and NKT10 cells that inhibit autoimmune diseases (173–175). Such diversity in function needs to be considered when targeting the adjuvant activities of iNKT cell agonists because, depending on the route of vaccinations, some subsets may have more influence on shaping anti-IAV immune responses than others, which could considerably affect the quality and the durability of protection. Another consideration is the extensive variability in iNKT cell subset ratios among inbred mouse strains and genetically outbred humans and animals, which can result in extensive variability in outcomes. Indeed different ratios of NKT1/NKT2 subsets are thought to underlie the dimorphic responses that α-GalCer treatment elicits in C57BL/6 and BALB/c mice for a host of different diseases (95). Currently, a unifying model linking iNKT cell transcription factors and functions is lacking for humans (and other species). Nevertheless, iNKT cells are probably comprised of functionally distinct subsets in all species that expresses these cells. Accordingly, the variability in iNKT cell subsets should be considered a potential source of variation in people or animals administered glycolipids to stimulate iNKT cell adjuvant activities.

### iNKT Cell Agonists

Since α-GalCer is the first iNKT cell ligand to be discovered and strongly activates iNKT cells, this agent and its synthetic derivative KRN7000 have been widely used to study the therapeutic potential of iNKT cells, including their adjuvant activities (18, 19, 176). α-GalCer is a glycosylceramide molecule, composed of an α-anomeric sugar linked to a 26-carbon-long fatty acid chain and an 18-carbon-long sphingosine base (15). While this glycolipid induces a mixed Th1/Th2 cytokine response, various structural analogs of α-GalCer that skew iNKT cell cytokine production toward a Th1 or Th2 response have been developed. These modifications include altering the length, saturation level, and branching of the alkyl and sphingosine chains, while other derivatives contain modifications at the glycosyl head (96, 97, 173, 177–181).

In general, α-GalCer analogs with truncated fatty acid chains or the addition of double bonds in the acyl chain, such as OCH ((2S,3S,4R)-1-*O*-(α-D-galactopyranosyl)-N-tetracosanoyl-2-amino-1,3,4-non-anetriol) and C20:2, skew iNKT cells toward producing Th2 cytokines (173, 177). In contrast, iNKT cells can be preferentially activated to produce Th1-like cytokines by (i) α-GalCer derivatives that contain a CH2 group in place of the glycosidic oxygen (180), (ii) α-C-GalCer analogs that contain an oxygen residue in the galactose sugar ring (182), and (iii) 7DW8-5 and C34 analogs that, respectively, possess methylene and aromatic residues inserted into their fatty acid chains (183, 184). Most studies on the adjuvant activities of iNKT cells use α-GalCer, which generates potent cellular and humoral immunity due to the mixed Th1/Th2 cytokines elicited. However, several Th1-inducing reagents, including 7DW8-5 and C-glycoside, substantially enhance vaccine responses against malaria, HIV, and IAV vaccines in mice (33, 180, 183). Some studies have reported that these agents are superior to α-GalCer for boosting vaccine-mediated immune responses due to a greater ability to trans-activate other immune cells, especially NK cells, to produce IFN-γ (92, 183).

The burgeoning list of synthetic iNKT cell ligands provides new opportunities to tune iNKT cell responses for a desired vaccine-supporting immune outcome. For IAV vaccines, the most promising iNKT cell-stimulating antigens are those which polarize mouse iNKT cells to produce Th1 cytokines or a mixture of Th1 and Th2 cytokines, which are naturally important for antiviral immune responses. Nevertheless, many of these reagents do not polarize human iNKT cells to the same degree as mouse iNKT cells due partially to the structural differences in mouse and human CD1d molecules that affect TCR/CD1d/antigen interactions (91, 92, 178). This is an important consideration for translating preclinical animal vaccine studies to humans.

### Vaccine Format

Studies with different disease models have shown that α-GalCer treatment can have diverse immune effects depending on the timing, route, and dose of α-GalCer administration. Understanding how these parameters affect the adjuvant activities of therapeutically activated iNKT cells is critical for optimizing iNKT cell-adjuvanted IAV vaccines. The timing of α-GalCer treatment is a concern for prime-boost vaccination strategies that administer more than one α-GalCer dose. This is because the strong activation from a primary vaccination may render iNKT cells hypo-responsive to an additional stimulation. Most studies on the adjuvant activities of iNKT cell agonists for IAV vaccines employ multiple vaccine applications (Table 1) even though iNKT cells are reported to remain anergic to restimulation within 3 months after the initial stimulation. These reports seldom assess whether increases in immune responses are from the effects of iNKT cells stimulation or from the vaccine antigen alone. However, it has been shown that the intramuscular delivery of α-GalCer avoids iNKT cell anergy in both mice and pigs (21, 30, 52, 153), suggesting that the risk of iNKT cell hypo-responsiveness may be low if prime-boost vaccination strategies were employed with IAV vaccines using the i.m. route.

Studies that have employed inactivated IAV vaccines, viral peptides, and DNA vaccines have used a variety of immunization routes, although intramuscular injection is the most common. In contrast, iNKT-adjuvanted live attenuated IAV vaccines have mostly been delivered intranasally to induce protective immunity at the site of infection. In general, the systemic routes of vaccine and α-GalCer co-administration globally activate iNKT cells in a way that greatly enhances neutralizing antibodies (21, 23, 30). However, this route is not as effective as intranasal administration at inducing secretory IgA antibodies necessary for cross-protection against heterologous virus strains (23). Studies that have compared the efficacy of different vaccination sites include the report by Galli et al. which showed that the intraperitoneal, subcutaneous, intramuscular, and intravenous immunization routes were equally effective and better than intranasal administration at inducing vaccine-specific antibodies (30). Another study showed that BALB/c mice intranasally vaccinated with α-GalCer in combination with HA antigen derived either from A/PR8 (H1N1) or A/Yamagata (H1N1) were protected from subsequent infection with A/PR8 (H1N1) live virus and that the A/Yamagata HA antigen vaccine induced anti-PR8 HA IgA and IgG antibodies. In contrast, the same vaccines administered by the intramuscular route failed to induce IgA antibodies and did not provide cross-protective immunity (23). The same study compared the effect of intraperitoneally and intranasally delivered α-GalCer on an intranasally delivered A/PR8 (H1N1) vaccine. Despite strongly activating splenic and hepatic iNKT cells, intraperitoneally delivered α-GalCer did not induce anti-PR8 IgA and IgG antibodies as a response to the A/PR8 HA antigen vaccine administered intranasally, indicating that iNKT cell ligands must be co-administered with viral antigens to enhance an intranasally delivered vaccine (23).

The adjuvant effects of glycolipid-stimulated iNKT cells enhance immune responses to a wide variety of vaccine formats. Such versatility stems from the powerful immunoregulatory properties of iNKT cells, which affect almost every branch of the immune system and which are generally more diverse than the immunomodulatory effects induced by traditional adjuvants. A drawback of this potency is that iNKT cell responses can reduce the efficacy of live attenuated virus vaccines by inducing antiviral host responses that eliminate the weakened vaccine virus before it has had a chance to induce adaptive immunity. Consequently, it will be necessary to carefully titrate each glycolipid ligand to find a dose that increases, rather than decreases, immunity against live attenuated virus vaccines. Dosage is less important for non-attenuated vaccines, although high doses of glycolipid ligands may render iNKT cells anergic to secondary activation.

## Potential Pitfalls

Several obstacles must be overcome before the immunomodulatory activities of iNKT cells can be used for human or livestock vaccines. Of paramount concern is the safety of this strategy as the potent cytokine responses generated by therapeutically activated iNKT cells can sometimes result in immunopathological inflammation and/or disease exacerbation (185–187). This includes reports that α-GalCer administration can induce acute airway hyper-reactivity in mice (188), non-human primates (189), and pigs (76). In addition, the co-administration of α-GalCer and the model antigen ovalbumin resulted in allergic airway inflammation in mice (190). These results should serve as a note of caution that combining α-GalCer with intranasally administered IAV vaccines could cause potentially life-threatening airway inflammation. Another concern is that α-GalCer-stimulated iNKT cells may increase the risk of vaccine-associated enhanced respiratory disease. This phenomenon occurs when inactivated IAV vaccines include a virus strain of the same hemagglutinin subtype as a subsequent challenge virus, but with substantial antigenic shift (191). This occasionally generates IgG antibodies that cross-react with the heterologous virus proteins but lack the ability to neutralize the heterologous virus effectively. These antibodies might instead bind epitopes in the HA stem region (HA2) of the heterologous virus, which facilitates infection of host cells by enhancing virus fusion activity (191). As iNKT cell activation boosts the size and the complexity of humoral responses, it is possible that they will also increase non-neutralizing antibody responses against heterologous viruses. Another concern is the potential that immunity from iNKT cell-adjuvanted vaccines will be inconsistent and unpredictable due to the high interindividual variability in iNKT cell frequency and function in genetically outbred species, including humans (192, 193). Indeed some studies have reported that only patients with high iNKT cell frequencies were likely to benefit from iNKT cell-based treatments (194, 195). It is currently difficult to predict whether an individual's iNKT cells will produce an immunogenic or tolerogenic response to stimulation, and the danger exists that iNKT cell activation might actually reduce the efficacy of IAV vaccines. This is further complicated by the phenomenon that iNKT cells undergo substantial age-related alterations in concentration and effector functions that are likely to impact their response to glycolipid antigens (196–200). Finally, it is important to consider that most studies in this field were conducted using mouse models which, although they have provided extensive knowledge about the fundamental role of iNKT cells during IAV infections, do not closely mirror humans for iNKT cell physiology. Furthermore, mice are not natural hosts of IAV infections and usually develop much more severe disease than humans when infected with mouse-adapted IAV strains (201). Thus, another obstacle is the need for more studies in valid preclinical animal models to translate iNKT cell-adjuvanted vaccination to the clinic.

## Concluding Remarks

Durable and broadly protective IAV vaccines are greatly needed to counteract the growing threats of morbidity, mortality, and economic losses from seasonal and pandemic IAV infections. Current vaccine formulations do not provide long-lasting and cross-protective immunity, partly because they do not induce sufficient T cell help from virus-specific T cells. iNKT cells may help to overcome this limitation because they can be uniformly and specifically activated by therapeutic glycolipid antigens to supply a universal form of T cell help capable of expanding virus-specific antibodies and CD8^+^ T cells. Nevertheless, significant hurdles remain before the adjuvant activities of iNKT cells can be utilized in humans and livestock for vaccines against IAV and other pathogens. Future research should focus on testing this approach using preclinical animal models with high human translational potential, such as swine and non-human primates. Such studies will help determine the translatability of iNKT cell-adjuvanted vaccines for other respiratory diseases which have shown promise in mouse models (202).

## Author Contributions

JD and JR contributed to the conceptualization, writing, review, and editing. DC, WG, and BA contributed to the writing. All authors contributed to the article and approved the submitted version.

## Conflict of Interest

The authors declare that the research was conducted in the absence of any commercial or financial relationships that could be construed as a potential conflict of interest.
